# ERBB4 as a therapeutic target in aortic dissection: Implications for cell-based therapies in vascular regeneration

**DOI:** 10.17305/bb.2025.11925

**Published:** 2025-03-14

**Authors:** Yu Shi, Jinjin Meng, Shengqiang Zhang, Shaofeng Yang, Ge Liu, Zhen Wu, Chongwen Shen, Chao Shi

**Affiliations:** 1 Continuing Education College, Anhui Medical University, Hefei, Anhui, China; 2 Department of Cardiovascular Surgery, The First Affiliated Hospital of Bengbu Medical University, Bengbu, Anhui, China

**Keywords:** Aortic dissection, AD, Erb-B2 receptor tyrosine kinase 4, ERBB4, vascular smooth muscle cells, VSMCs, cell-based therapy, NF-κB pathway

## Abstract

This study investigates the role of Erb-B2 receptor tyrosine kinase 4 (ERBB4) in aortic dissection (AD) pathogenesis and its potential as a therapeutic target in cell-based therapies. Using immunohistochemical (IHC) staining, we examined the phenotypic transition of vascular smooth muscle cells (VSMCs) in thoracic aortic tissue from AD patients. RNA sequencing identified differentially expressed genes (DEGs), highlighting ERBB4 as a key regulator. *In vitro*, ERBB4 knockdown in human aortic smooth muscle cells (HASMCs) was assessed using cell counting kit-8 (CCK-8 assays), wound healing, transwell migration, colony formation, flow cytometry, and tube formation assays. Our results showed significant inhibition of cell viability, migration, proliferation, and angiogenesis. *In vivo*, ERBB4 knockdown reduced inflammatory cell infiltration and enhanced collagen fiber contractility, as demonstrated by Masson staining. Mechanistically, ERBB4 silencing suppressed integrin-binding proteins (CD151, ITGAE, ITGB5) and inhibited NF-κB signaling (p-IκBα, p-NF-κB-65). These findings suggest that ERBB4 is critical in AD progression and that its knockdown mitigates pathological changes in HASMCs, reduces inflammation, and restores collagen contractility. ERBB4 may serve as a promising target for cell-based therapies aimed at restoring vascular integrity and function, providing potential new treatment strategies for AD patients.

## Introduction

Aortic dissection (AD) is a life-threatening cardiovascular emergency with high incidence and mortality rates. Without timely intervention, mortality increases by 1%–2% per hour within the first 48 h, reaching up to 90% within a week [1, 2]. Currently, surgical intervention is the only life-saving treatment, as no effective pharmaceutical options exist [3]. Despite extensive research, the precise pathophysiological mechanisms of AD remain poorly understood.

In recent years, the incidence of AD has been increasing, making it a key research focus in cardiovascular disease. Its occurrence is influenced by both genetic and environmental factors, with gene mutations playing a significant role in its pathogenesis. Identifying effective molecular targets is therefore crucial for advancing AD treatment strategies.

Erb-B2 receptor tyrosine kinase 4 (ERBB4), also known as human epidermal growth factor receptor 4 (HER4), is a member of the epidermal growth factor receptor (EGFR) family [4]. This family plays a crucial role in various biological processes, including cell growth, proliferation, differentiation, and apoptosis [5, 6]. Additionally, ERBB4 has been linked to inflammatory pain in dorsal root ganglion sensory neurons [7]. The ERBB4 gene encodes a protein involved in signaling pathways essential for heart development, angiogenesis, and vascular wall homeostasis. Variations or abnormal expression of ERBB4 may disrupt these processes, potentially compromising aortic structure and function and increasing the risk of AD. However, the precise mechanisms by which ERBB4 contributes to AD pathogenesis remain unclear and require further investigation.

This study aims to identify differentially expressed genes (DEGs) in Alzheimer’s disease and elucidate the relationship between the hub gene ERBB4 and AD using molecular biology, cell biology, and genetic approaches. We analyzed ERBB4 expression in AD patients and explored its specific role in disease development and progression. By investigating the underlying mechanisms, this study seeks to provide new insights into AD prevention, diagnosis, and treatment.

This study aims to identify DEGs in Alzheimer’s disease and elucidate the relationship between the hub gene ERBB4 and AD using molecular biology, cell biology, and genetic approaches. We analyzed ERBB4 expression in AD patients and explored its specific role in disease development and progression. By investigating the underlying mechanisms, this study seeks to provide new insights into AD prevention, diagnosis, and treatment.

## Materials and methods

### Tissues sample

Aortic tissue samples were obtained from three patients undergoing Stanford type A dissection surgery, while control aortic tissue (Control) was collected from the aortas of coronary artery bypass patients (*n* ═ 3). Each sample was divided into two parts: one portion was stored at −80 ^∘^C for western blot analysis, and the other was fixed in a 4% paraformaldehyde solution for further examination. This study adhered to the principles of the Declaration of Helsinki and was approved by the Ethics Committee of the First Affiliated Hospital of Bengbu Medical University.

### Immunohistochemical (IHC) staining

IHC analysis was performed on aortic tissue. Samples were treated with 3% hydrogen peroxide for 10 min, rinsed with phosphate-buffered saline (PBS), and blocked with 10% normal goat serum in PBS for 1 h at 37 ^∘^C. Sections were then incubated overnight at 4 ^∘^C with primary antibodies, followed by the appropriate secondary antibody and DAB staining (Beyotime, Shanghai, China). Finally, the sections were sealed with gum.

### RNA sequencing

RNA sequencing was conducted by Seqhealth Technology Co., Ltd. (Wuhan, China). Briefly, total RNA was extracted from human aortic tissue samples using TRIzol Reagent (Invitrogen, Cat. No. 15596026, USA). Stranded RNA sequencing libraries were prepared from 2 µg of total RNA using the KCTM Stranded mRNA Library Prep Kit for Illumina^®^ (Wuhan Seqhealth Co., Ltd., China), following the manufacturer’s instructions. PCR products were enriched, quantified, and sequenced on a DNBSEQ-T7 sequencer (MGI Tech Co., Ltd., China) using the PE150 model. DEGs were identified using the edgeR package (version 3.12.1), with a fold-change cutoff of 2 and a *P* value threshold of 0.05 for statistical significance. Gene Ontology (GO) and Kyoto Encyclopedia of Genes and Genomes (KEGG) enrichment analyses for DEGs were performed using KOBAS software (version 2.1.1).

### Cell culture and transfection

Human aortic smooth muscle cells (HASMCs) were obtained from the American Type Culture Collection (ATCC, USA) and cultured in Dulbecco’s Modified Eagle’s Medium (DMEM) supplemented with 10% fetal bovine serum (FBS; Gibco, USA), 100 U/mL penicillin, and 100 µg/mL streptomycin. The cells were maintained at 37 ^∘^C with 5% CO_2_. In the model group, HASMCs were cultured in high-glucose DMEM. Three siRNAs were designed by Ribobio (Guangzhou, China) and transfected into HASMCs using a siRNA transfection kit (Thermo Fisher Scientific, USA), following the manufacturer’s instructions.

### Cell counting kit-8 (CCK-8)

After transfection, the cells were evenly seeded into a 96-well plate at a density of 2000 cells per well and cultured for 72 h. The culture medium was then discarded, and CCK-8 detection reagent was added. The cells were incubated at 37 ^∘^C for 1 h, and the optical density at 450 nm was measured using a microplate reader.

### Wound healing assay

HASMCs were seeded into a six-well plate and cultured until reaching approximately 80%–90% confluency. A linear wound was then created in the center of each well using a sterile 200 µL pipette tip. Debris was removed by washing the wells twice with PBS, after which the cells were incubated in DMEM containing 1% FBS to minimize proliferation. Treatments—including the vehicle control, growth factors, or pharmacological inhibitors—were added as indicated. Images of the wound area were captured at 0, 24, and 48 h using an inverted microscope equipped with a camera. The distance between the advancing wound edges was measured using ImageJ software (USA), and data were expressed as the percentage of wound closure relative to the initial wound area.

### Transwell assay

The transfected cells were collected, resuspended in serum-free medium, and added to the upper chamber. DMEM high-glucose medium containing 20% FBS (Hyclone, USA) was added to the lower chamber. After 24 h of incubation, the medium was discarded, and the cells were fixed with methanol for 30 min. The cells were then stained with 0.1% crystal violet (MedChemExpress, USA) for 20 min. Non-migrated cells on the upper chamber were gently removed with a cotton swab. Finally, the migrated cells were observed and photographed under an optical microscope.

### Flow cytometry

Following a 48-h transfection period, cells from each experimental group were harvested, fixed in absolute ethanol, and resuspended in centrifuge tubes. A total of 2.0 × 10^5^ cells were collected and subsequently treated with 500 µL of propidium iodide (PI) staining solution (Abcam, UK) for 30 min in the dark. Flow cytometry (BD, USA) was then used to analyze the cell cycle.

### Tube formation assay

The formation of capillary-like structures *in vitro* was assessed using the standard Matrigel assay. HASMCs were seeded at a density of 2 × 10^4^ cells per well in 24-well plates with different treatments in serum-free media. The plates were pre-coated with growth factor-reduced Matrigel matrix (BD Biosciences, USA) and incubated at 37 ^∘^C for 12 h. Tube formation was quantified by analyzing four random fields at 200× magnification per well and comparing the results to untreated control wells.

### Animal model

A total of 10 male Sprague-Dawley (SD) rats (three weeks old) were purchased from The Shanghai Slake Laboratory Animal Co., Ltd (China). After a seven-day adaptive feeding period, an aortic dissection model was induced by administering β-aminopropionitrile via gavage at a dosage of 1 g/kg/day for six weeks, until the formation of aortic dissection. Rats that did not successfully develop the model were excluded from the study after experimentation. Successful modeling criteria included survival during drug administration, followed by anesthesia, euthanasia via cervical dislocation, and extraction of the aorta for pathological analysis. The animals were then divided into two groups: shCtrl and shERBB4. Each rat received a 200 µL injection of adenovirus (designed by Ribobio) via the tail vein. The adenovirus was administered two days before β-aminopropionitrile treatment and then once every two weeks. The occurrence of aortic dissection was confirmed by the presence of a significant false lumen containing numerous red blood cells.

### HE staining

Aortic specimens were fixed overnight in 4% paraformaldehyde, then dehydrated, embedded in paraffin, and sectioned into 5 µm slices. After deparaffinization and rehydration, the sections were stained with H&E (Solarbio, Beijing, China), dehydrated with alcohol, cleared with xylene, cover-slipped, and examined under an optical microscope. Images were captured and recorded.

### Masson staining

The aortic tissue samples were deparaffinized and stained using Masson’s trichrome (Beyotime, Shanghai, China). Subsequently, images of five microscopic fields were captured at 200× magnification using an optical microscope (Olympus, Japan).

### Elastic Verhoeff-Van Gieson (EVG) staining

The aortic tissue was cut into 5 µm sections, then dewaxed and hydrated. EVG staining was applied for 30 min, followed by background differentiation and counterstaining with EVG (Solarbio, Beijing, China). After rinsing with distilled water, the sections were mounted and examined under a microscope for image acquisition and analysis.

### Quantitative real time-PCR (qRT-PCR)

Trizol reagent (Thermo Fisher Scientific, USA) was added to the collected cells to extract total RNA. The extracted RNA was then reverse transcribed into cDNA using a reverse transcription kit. qPCR was performed using cDNA as a template and a SYBR qPCR kit (Beyotime, Shanghai, China). GAPDH served as an internal reference, and gene expression levels were calculated using the 2^−ΔΔCT^ method.

### Western blot

Cells from each experimental group were harvested and lysed using Radioimmunoprecipitation Assay (RIPA) buffer (Beyotime, Shanghai, China) to extract total cellular protein. Protein concentration was determined using the bicinchoninic acid (BCA) assay (Beyotime, Shanghai, China). Equalized protein samples were separated via sodium dodecyl sulfate-polyacrylamide gel electrophoresis (SDS-PAGE) and transferred onto a polyvinylidene difluoride (PVDF) membrane (Millipore, USA). To prevent non-specific binding, membranes were blocked with 5% non-fat milk in Tris-buffered saline containing 0.1% Tween-20 (TBST) for 1 h at room temperature, followed by overnight incubation at 4 ^∘^C with primary antibodies against ERBB4 (1:1000, Proteintech, Wuhan, China), α-actin (1:1000, Proteintech), Transgelin (TAGLN) (1:1000, Elabsience, Shanghai, China), SPP1 (1:1000, ZENBIO, Chengdu, China), epiregulin (1:1000, Biorbyt, UK), CD151 (1:1000, Proteintech), ITGAE (1:1000, ab224202, Abcam, UK), ITGB5 (1:1000, Proteintech), p-IκBα (1:5000, Proteintech), IκBα (1:5000, Proteintech), p-NF-κB-65 (1:2000, Proteintech), NF-κB-65 (1:1000, Proteintech), and GAPDH (1:50000, Proteintech) as a loading control. After incubation, membranes were washed three times with TBST and incubated with a horseradish peroxidase (HRP)-conjugated secondary antibody (1:10000, Absin, Shanghai, China) for 1 h at room temperature. Following another three TBST washes, protein bands were detected using an enhanced chemiluminescence (ECL) system, and their intensities were quantified by densitometry.

### Ethical statement

All experiments performed in this study adhered to the ethical guidelines of the Declaration of Helsinki and were approved by the Ethics Committees of Anhui Medical University (No. 2024253). No patients participated in this study.

### Statistical analysis

Data from multiple experimental repeats were collected and statistically analyzed using GraphPad Prism 8.0. Differences between experimental groups were compared using Student’s *t*-test.

## Results

### Phenotype transition of vascular smooth muscle cells (VSMCs) in aortic dissection patients

**Figure 1.**
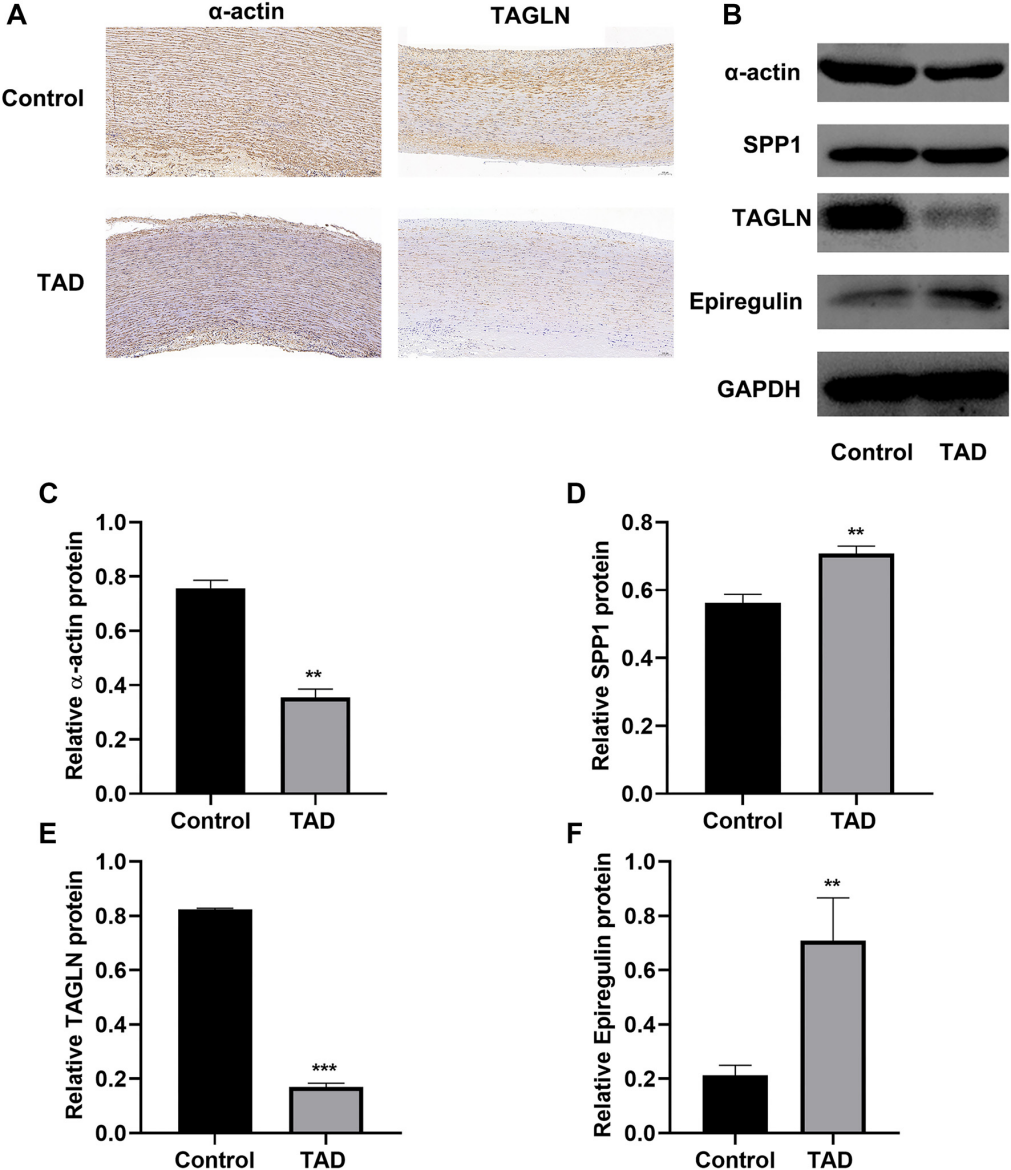
**Phenotype transition of VSMC cells.** (A) IHC results revealed the decreased levels of α-actin, and TAGLN in aortic tissue; (B) Western blot results presented the decreased levels of α-actin, SPP1, TAGLN, and the highly expressed of epiregulin in aortic tissue. Bar graphs presented the quantification of α-actin (C), SPP1 (D), TAGLN (E), and epiregulin (F). vs Control group, **P < 0.01, ***P < 0.001. VSMC: Vascular smooth muscle cell; IHC: Immunohistochemical; TAGLN: Transgelin.

IHC results revealed that α-actin and TAGLN expression were reduced in the thoracic aortic dissection (TAD) group. Western blot analysis further confirmed these findings. Additionally, western blot results showed that the expression levels of SPP1 and epiregulin were increased in the TAD group compared to the control group (Figure 1, *P* < 0.05).

### DEGs were analyzed by RNA sequencing in the control and TAD group

**Figure 2.**
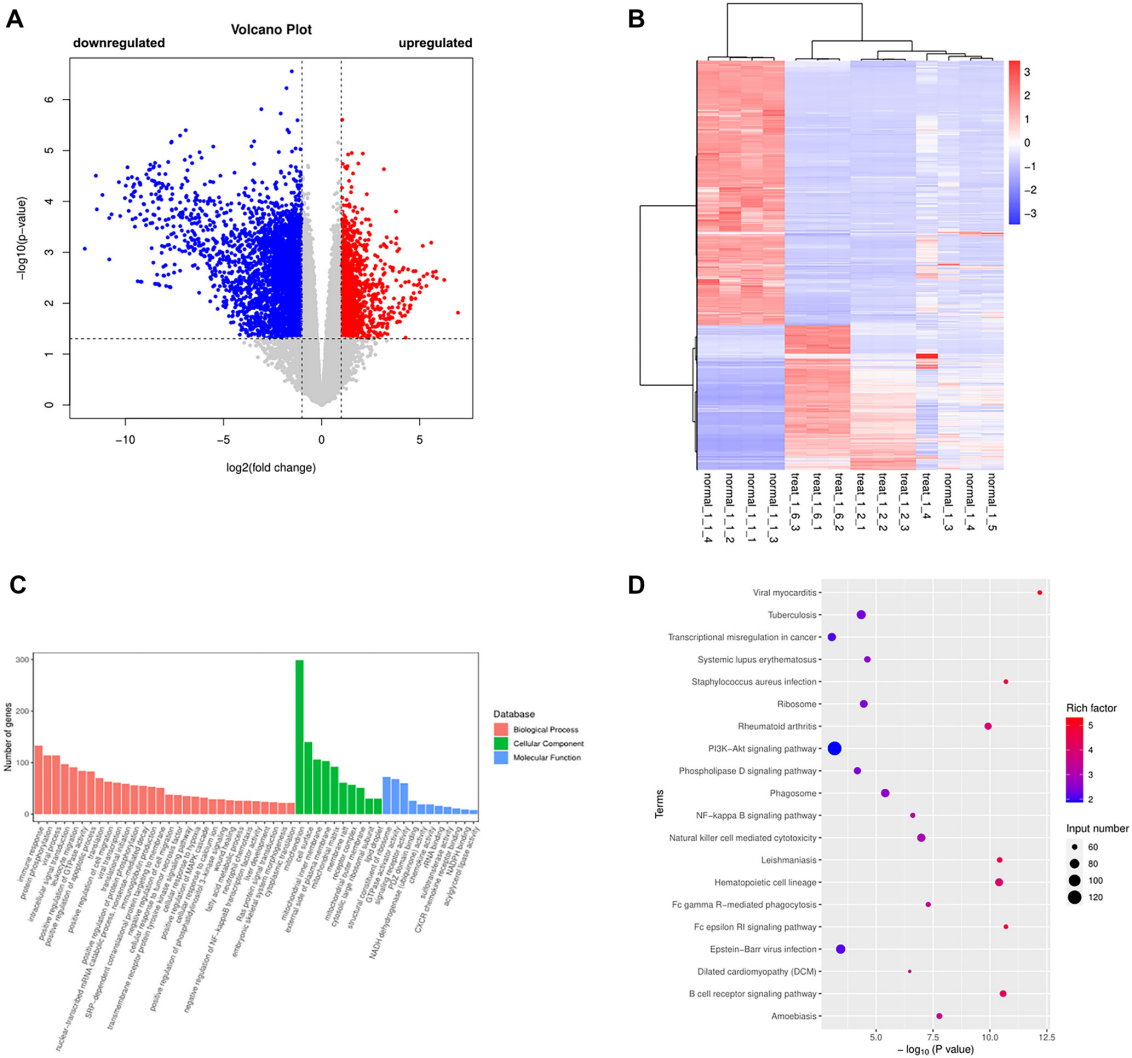
**DEGs were analyzed byRNA sequencing in the Control and TAD group.** (A) Volcano plots is created using the fold change and *P* adjust values, red presents upregulated genes, blue presents downregulated genes; (B) Cluster analysis of DEGs in the Control and TAD group; (C) GO enrichment analysis; (D) KEGG enrichment analysis. KEGG: Kyoto Encyclopedia of Genes and Genomes; GO: Gene Ontology; TAD: Thoracic aortic dissection; DEG: Differentially expressed gene.

RNA sequencing results showed 1878 upregulated genes and 3440 downregulated genes (Figure 2A and 2B). GO enrichment analysis revealed that the DEGs) were primarily enriched in leukocyte migration (GO:0050900), viral transcription (GO:0019083), SRP-dependent cotranslational protein targeting to the membrane (GO:0006614), integrin binding (GO:0005178), focal adhesion (GO:0005925), and lamellipodium formation (GO:0030032) (Figure 2C). Similarly, KEGG enrichment analysis indicated that the DEGs were mainly associated with pathways, such as Phagosome (hsa04145), Amoebiasis (hsa05146), Dilated cardiomyopathy (hsa05414), Fc gamma R-mediated phagocytosis (hsa04666), and the NF-kappa B signaling pathway (hsa04064) (Figure 2D).

### ERBB4 was chose for hub genes

**Figure 3.**
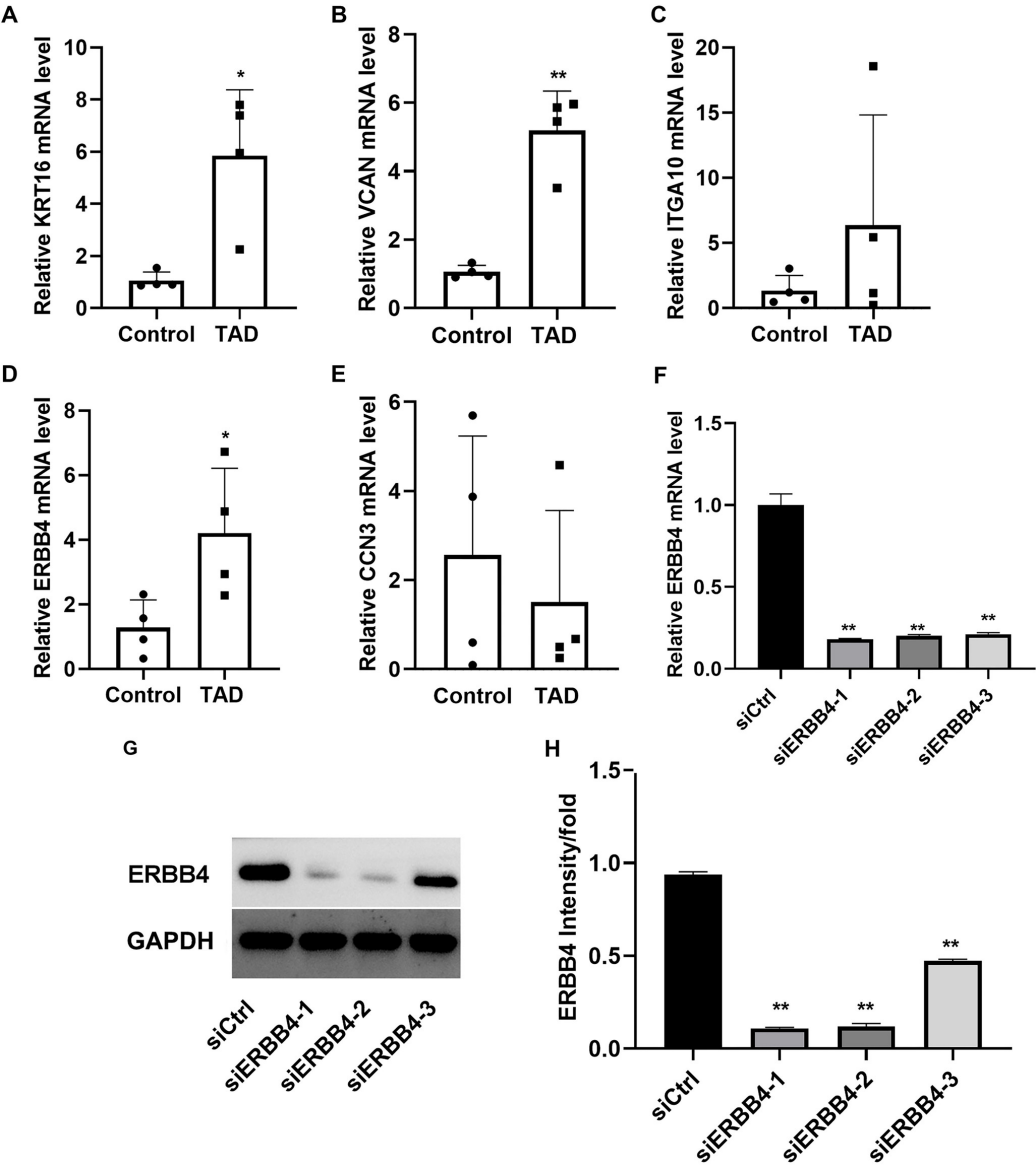
**ERBB4 was chose for hub genes.** The expression of KRT16 (A), VCAN (B), ITGA10 (C), ERBB4 (D), and CCN3 (E) mRNA levels were detected by qRT-PCR; (F) The efficiency of ERBB4 siRNA was detected by qRT-PCR; (G) Western blot result presented the efficiency of ERBB4 siRNA; (H) Bar graph presented the quantification of western blot results. *P < 0.05, **P < 0.01. ERBB4: Erb-B2 receptor tyrosine kinase 4; VCAN: Versican; qRT-PCR: Quantitative real time-PCR.

We analyzed the mRNA expression levels of KRT16, VCAN, ITGA10, ERBB4, and CCN3. Among them, KRT16, Versican (VCAN), and ERBB4 were significantly upregulated in the TAD group compared to the Control group (Figure 3A–3E, *P* < 0.05). Based on these results, we selected ERBB4 for further experiments. To suppress ERBB4 expression, we designed three siRNAs. qRT-PCR and Western blot analyses confirmed a significant reduction in ERBB4 expression following siRNA transfection. Among the three, siRNA-2 exhibited the highest knockdown efficiency. Therefore, we used siRNA-2 for all subsequent experiments (Figure 3F–3H, *P* < 0.05).

### ERBB4 knockdown suppressed HASMCs proliferation

**Figure 4.**
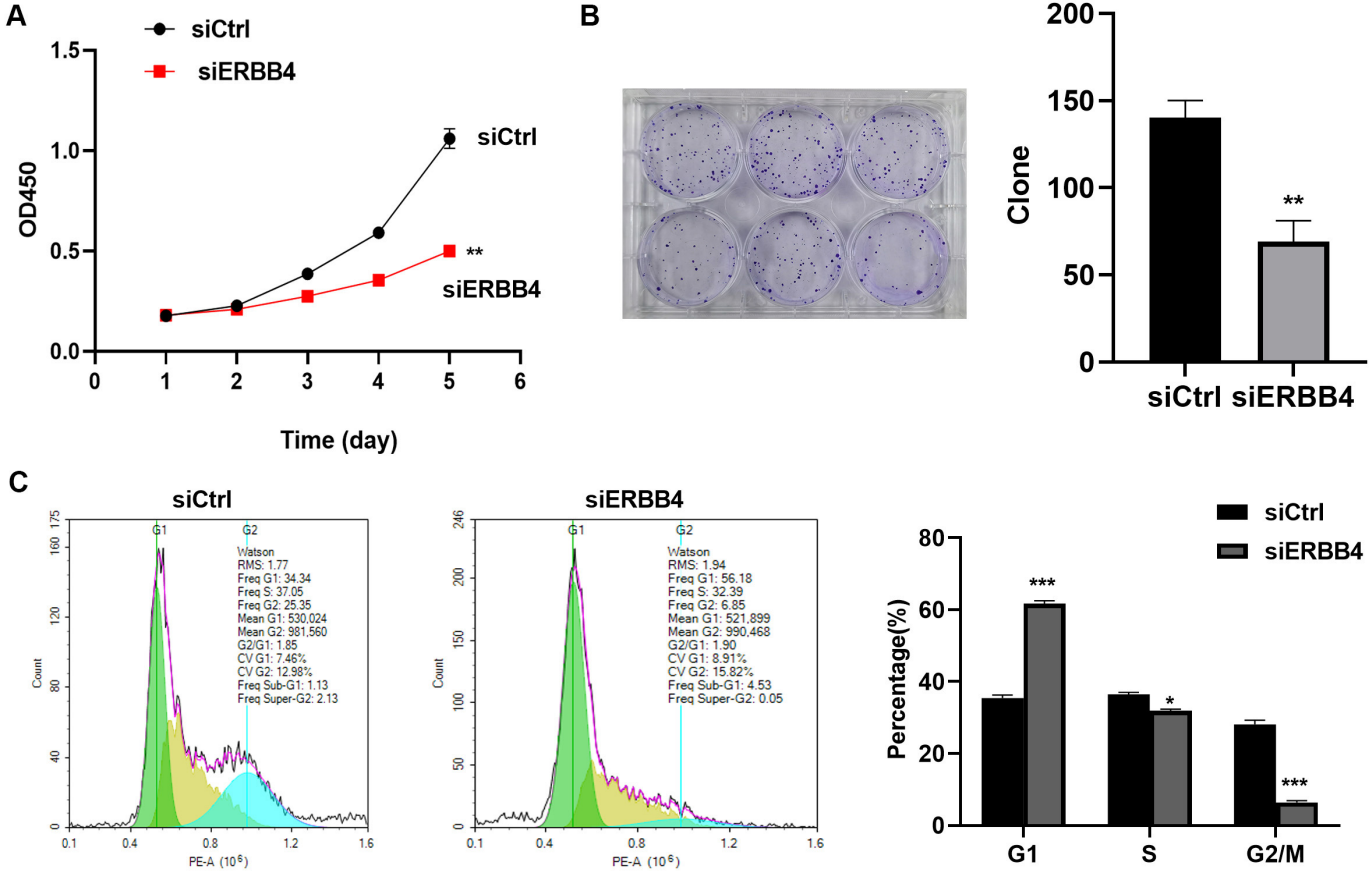
**ERBB4 knockdown suppressed HASMCs proliferation.** (A) CCK-8 results presented the inhibition of HASMCs viability after ERBB4 knockdown; (B) Clone formation assay presented the suppression of HASMCs proliferation after ERBB4 knockdown; (C) Flow cytometry confirmed the ERBB4 knockdown suppressed cells proliferation. vs siCtrl group, *P < 0.05, **P < 0.01, ***P < 0.001. ERBB4: Erb-B2 receptor tyrosine kinase 4; HASMC: Human aortic smooth muscle cell; CCK-8: Cell counting kit-8.

CCK-8 results revealed that cell viability was inhibited in the siERBB4 group compared to the siCtrl group (Figure 4A, *P* < 0.05). Colony formation assay results showed that cell proliferation was suppressed in the siERBB4 group relative to the siCtrl group (Figure 4B, *P* < 0.05). Cell cycle analysis indicated a decrease in the number of cells in the S phase and cell cycle arrest at the G1/S or G2/M checkpoints in the siERBB4 group. These findings suggest that ERBB4 knockdown suppresses cell proliferation (Figure 4C, *P* < 0.05).

### ERBB4 knockdown suppressed HASMCs migration and tube formation

**Figure 5.**
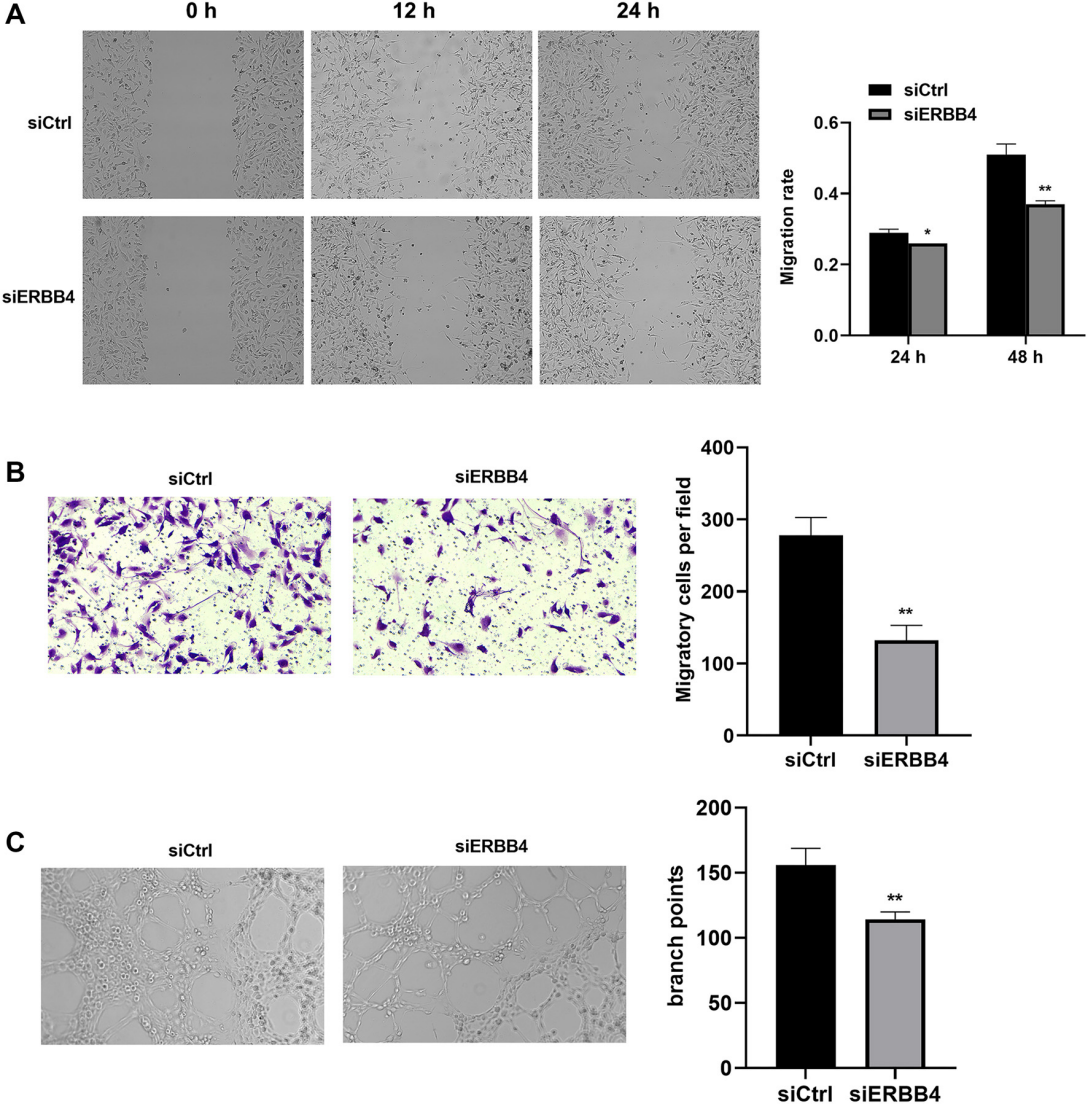
**ERBB4 knockdown suppressed HASMCs migration and tube formation.** Wound scratching healing (A) and transwell (B) assay revealed the suppression of HASMCs migration after ERBB4 knockdown; (C) Tube formation assay presented the inhibition of HASMCs angiogenesis after ERBB4 knockdown. vs siCtrl group, *P < 0.05, **P < 0.01. ERBB4: Erb-B2 receptor tyrosine kinase 4; HASMC: Human aortic smooth muscle cell.

The Transwell and wound scratch healing assays indicated that cell migration was diminished in the siERBB4 group compared to the siCtrl group (Figure 5A, B; *P* < 0.05). Tube formation capacity in HASMCs was evaluated based on the number of tubes and branch length. The results showed that HASMCs in the siCtrl group exhibited typical tube formation, whereas tube formation was significantly inhibited in the siERBB4 group (Figure 5C; *P* < 0.05).

### ERBB4 knockdown ameliorated pathological changes of aortic tissue

**Figure 6.**
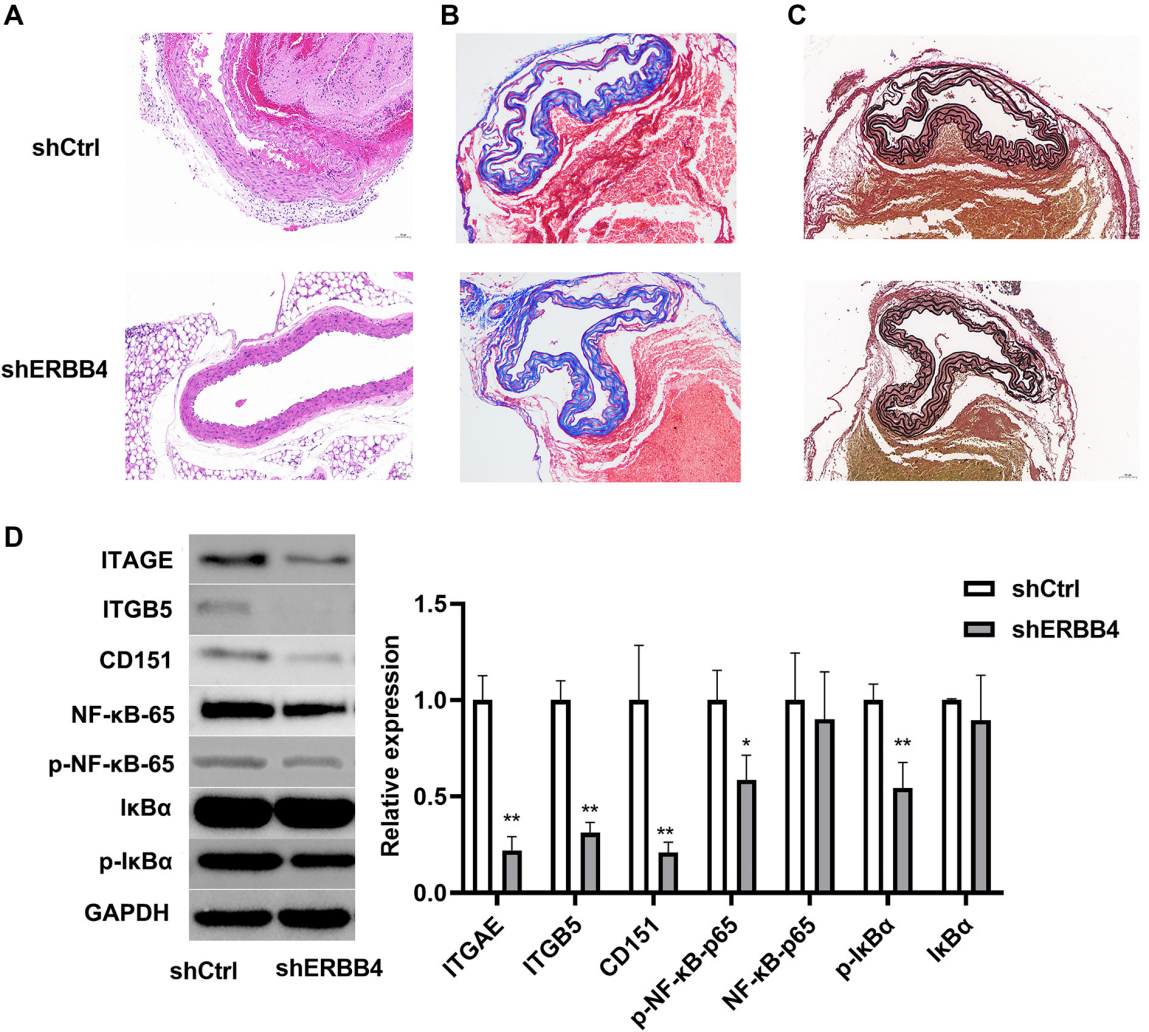
**ERBB4 knockdown improved pathological changes *in vivo*.** (A) HE staining presented the pathological changes in aorta tissues (scale bar: 50 µm); (B) Masson staining (scale bar: 50 µm); (C) Elastic van Gieson (EVG) staining presented the elastic fibers in AD (scale bar: 50 µm); (D) Western blotting results presented the down-regulation of integrin binding and NF-kappa B signaling pathway after ERBB4 knockdown. vs shCtrl group, *P < 0.05, **P < 0.01. ERBB4: Erb-B2 receptor tyrosine kinase 4; AD: Aortic dissection.

HE staining of aortic tissue revealed a large false lumen filled with numerous red blood cells, confirming the presence of an aortic dissection. In the shCtrl group, aortic wall inflammation was significantly increased, whereas inflammatory cell infiltration was reduced in the shERBB4 group (Figure 6A). Masson staining showed a significant increase in the contractile strength of collagen fibers in the shERBB4 group compared to the shCtrl group (Figure 6B). Additionally, in the shCtrl group, elastic fibers were increased but sparsely arranged and locally broken. In contrast, the shERBB4 group exhibited dense, regularly arranged elastic fibers, although some remained locally broken (Figure 6C).

### The underlying mechanism of ERBB4 was related to integrin binding and NF-kappa B signaling pathway

According to GO enrichment analysis (GO:000517 and GO:00018, *P* < 0.05), we examined the protein expression levels of integrin-binding molecules (CD151, ITGAE, ITGB5) and key components of the NF-κB signaling pathway (p-IκBα, IκBα, p-NF-κB-65, NF-κB-65) in tissue samples. Western blot results showed that the expression levels of CD151, ITGAE, ITGB5, p-IκBα, and p-NF-κB-65 were significantly reduced in the shERBB4 group (*P* < 0.05; Figure 6D).

## Discussion

The aorta, the largest blood vessel in the human body, is critically affected by disruptions to its structural integrity, leading to a pathological condition known as AD [8]. Vascular smooth muscle cells (VSMCs) play a key role in maintaining aortic wall stability, primarily through their ability to undergo a phenotypic switch [9, 10]. Under specific stimuli, VSMCs transition from a quiescent, contractile phenotype to a synthetic, proliferative, and migratory state [11]. This transformation is marked by decreased expression of contractile markers, increased synthesis of extracellular matrix (ECM) proteins, and heightened elastase production [12]. While this phenotypic plasticity enables VSMCs to respond to vascular injury and hemodynamic stress [13, 14], its dysregulation significantly contributes to disease progression [15–17]. In this study, we investigated the effect of ERBB4 on HASMCs isolated from the aorta.

Markers such as smooth muscle α-actin indicate a contractile phenotype, whereas osteopontin (OPN) and epiregulin are associated with a proliferative phenotype [18]. TAGLN, also known as SM22, is an actin-binding protein that regulates cytoskeletal dynamics and enhances cellular contraction [19–22]. In our study, we observed a significant downregulation of smooth muscle α-actin and TAGLN in TAD, while OPN and epiregulin levels were elevated. These findings confirm a phenotypic switch in VSMCs from a contractile to a proliferative and migratory state, highlighting their role in AD progression.

To identify novel therapeutic targets for TAD, we conducted RNA sequencing, revealing 1878 upregulated and 3440 downregulated genes in TAD tissues compared to controls. GO enrichment analysis highlighted pathways related to leukocyte migration, structural ribosomal components, and translational initiation. KEGG pathway analysis implicated viral myocarditis, Staphylococcus aureus infection, and B-cell receptor signaling. Among the DEGs, we selected KRT16, VCAN, ITGA10, ERBB4, and CCN3 for further validation using qRT-PCR. The results showed that VCAN, KRT16, and ERBB4 were significantly upregulated in TAD tissues. KRT16, located on chromosome 17q21.2, encodes the type I cytoskeletal protein CK16, typically expressed in various epithelial tissues. VCAN is a large extracellular proteoglycan highly expressed in the aorta. A recent study demonstrated that VCAN contributes to aortic disease in Marfan syndrome (MFS) by triggering Nos2 through Akt activation *in vivo* [23]. ERBB4, a member of the EGFR) family, has been implicated in various diseases [24–26]; however, its role in AD remains unexplored. Based on our preliminary screening and the gene’s potential significance, we selected ERBB4 for further investigation. To explore its role and mechanism, we used siRNA to suppress ERBB4 expression in high-glucose-cultured HASMCs. Among the tested siRNAs, siRNA-2 showed the highest knockdown efficiency and was used in subsequent experiments. Functional assays revealed that ERBB4 knockdown significantly reduced HASMC viability, proliferation, and migration. Flow cytometry further confirmed suppressed cell proliferation, indicated by a reduction in S-phase cell numbers and cell cycle arrest at the G1/S or G2/M checkpoints.

Previous studies have shown that neuregulin (NRG2) promotes cell proliferation in the subventricular zone via ERBB4 activation [27], while ERBB4-mediated vasculogenic mimicry has been reported in breast cancer [26]. Consistent with these findings, our study demonstrated that ERBB4 knockdown inhibited tube formation in HASMCs, suggesting a similar role in promoting angiogenesis in AD. *In vivo* experiments revealed that ERBB4 knockdown suppressed inflammatory cell infiltration in the aortic wall, increased collagen fiber contractility, and enhanced the density of elastic fibers. A healthy endothelium is crucial for maintaining normal vasodilation and blood flow regulation. By reducing inflammation, blood vessels can better respond to physiological stimuli, potentially improving circulation. Collagen, a major component of the aortic ECM, is essential for withstanding hemodynamic forces. Enhanced collagen contractility may increase the aorta’s resistance to dilation and rupture. Likewise, elastic fibers play a key role in preserving aortic wall integrity. In summary, our study conclusively demonstrated that ERBB4 knockdown effectively ameliorates AD phenotypes in an *in vivo* experimental model.

A recent study demonstrated that Costunolide can mitigate inflammation, VSMC apoptosis, and MMP2/9 levels while bolstering ECM integrity in TAD by suppressing the NF-κB signaling pathway [28]. Additionally, other signaling pathways are involved in AD [29, 30]. To further investigate the downstream mechanisms, we performed GO and KEGG enrichment analyses, focusing on proteins associated with integrin binding (e.g., CD151, ITGAE, ITGB5) and the NF-κB signaling pathway. Western blot analysis revealed significant reductions in CD151, ITGAE, ITGB5, p-IκBα, and p-NF-κB-65 expression following ERBB4 knockdown, suggesting that ERBB4 modulates AD progression through these pathways. However, our study has several limitations. First, since AD progresses over a long period, the long-term impact of ERBB4 on AD requires further investigation. Second, *in vivo* models may not fully capture the complexity of human AD, which could limit the applicability of our findings. Third, the delivery systems and potential off-target effects of ERBB4 knockdown must be carefully considered, as they are crucial for ensuring the safety and efficacy of ERBB4-related gene therapies in the future.

## Conclusion

Our study demonstrates that ERBB4 is highly expressed in AD and plays a critical role in promoting VSMC phenotypic transition, contributing to AD development. ERBB4 knockdown mitigates these pathological changes, helping to preserve vascular integrity. These findings suggest that ERBB4 could be a promising therapeutic target for AD. Identifying ERBB4’s role provides new insights into the underlying pathological mechanisms of AD. Future preclinical and clinical studies are needed to further validate the efficacy and safety of targeting ERBB4 in AD patients.

## Data Availability

The data used to support the findings of this study are available from the corresponding author upon request.
